# Can prospective systematic reviews of animal studies improve clinical translation?

**DOI:** 10.1186/s12967-019-02205-x

**Published:** 2020-01-09

**Authors:** Pandora Pound, Merel Ritskes-Hoitinga

**Affiliations:** 1 Safer Medicines Trust, PO Box 122, Kingsbridge, TQ7 9AX UK; 2 SYRCLE, Department for Health Evidence, Radboud University Medical Center, PO Box 9101, Route 133, 6500 HB Nijmegen, The Netherlands

**Keywords:** Systematic review, Preclinical, Animal research, Translation

## Abstract

Systematic reviews are powerful tools with the potential to generate high quality evidence. Their application to animal studies has been instrumental in exposing the poor quality of these studies, as well as a catalyst for improvements in study design, conduct and reporting. It has been suggested that prospective systematic reviews of animal studies (i.e. systematic reviews conducted prior to clinical trials) would allow scrutiny of the preclinical evidence, providing valuable information on safety and efficacy, and helping to determine whether clinical trials should proceed. However, while prospective systematic reviews allow valuable scrutiny of the preclinical animal data, they are not necessarily able to reliably predict the safety and efficacy of an intervention when trialled in humans. Consequently, they may not reliably safeguard humans participating in clinical trials and might potentially result in lost opportunities for beneficial clinical treatments. Furthermore, animal and human studies are often conducted concurrently, which not only makes prospective systematic reviews of animal studies impossible, but suggests that animal studies do not inform human studies in the manner presumed. We suggest that this points to a confused attitude regarding animal studies, whereby tradition demands that they precede human studies but practice indicates that their findings are often ignored. We argue that it is time to assess the relative contributions of animal and human research in order to better understand how clinical knowledge is actually produced.

## Introduction

Systematic reviews (SRs) are powerful tools with the potential to generate high quality evidence. Their application to animal studies has exposed the poor quality of the majority of these studies, highlighting that many essential procedures such as randomisation and blinding are frequently not performed or reported [1, 2]. SRs have also been a catalyst for improvements in the design, quality and reporting of animal studies [3, 4]. As unreliable data cannot be used to draw any reliable conclusions, SRs can eventually lead (as a result of transparency encouraging methodological improvements) to more reliable and useful data, as has been the case with clinical trials. SRs use already available data (producing new scientific information without using more animals) and can prevent the unnecessary duplication of animal experiments by establishing the status of a body of evidence in a field. Since commencing SRs of animal studies, for example, Radboud University in the Netherlands has seen a 35% drop in animals used [5]. Consequently, SRs have value in terms of reducing research waste and promoting the ‘reduce’ component of the 3Rs [6]. SRs of high-quality animal studies may also provide new insights and enable scientific decisions to be evidence-based. For example, they have potential to complement ongoing work to select optimal animal models [7–9] by directing researchers towards those that are most predictive, or they may direct researchers away from animal models altogether.

Can SRs of animal studies also improve clinical translation? Certainly they have potential to do so, as they can make the evidence obtained from animal studies more transparent and accessible. They are able to synthesise and summarise large bodies of evidence, identify knowledge gaps and produce definitive answers based on all the available evidence, removing the need to try and make sense of the sometimes contradictory findings that emerge from individual studies within a body of research. They are also able to throw light on the validity of the data emerging from preclinical animal studies, which is of key importance to those considering clinical trials. Consequently, many (including ourselves) have advocated that SRs of animal studies should be routinely conducted on a prospective basis, i.e. prior to commencing clinical trials [3, 4, 10–19]. However, at present most SRs of animal studies are conducted retrospectively, after the corresponding clinical trials have taken place.

### What can retrospective SRs of animal studies tell us about the potential of prospective SRs?

In cases where clinical trials have either found no treatment effects, or harmful effects, SRs of animal studies are sometimes conducted retrospectively in an attempt to understand why the clinical trials went ahead. For example, after Horn et al’s SR of clinical trials of nimodipine for acute stroke found no evidence of a clinically important effect [20], the authors systematically reviewed the relevant animal studies [21] and concluded that these had not provided convincing evidence of benefit to support the decision to proceed to clinical trials. This was also the case for low level laser therapy for wound healing; after a SR of human studies found that the treatment was ineffective in humans [22], the authors systematically reviewed the animal studies [23] and concluded that they had not provided unequivocal evidence to substantiate the decision to conduct clinical trials. Similar conclusions were drawn in the cases of fluid resuscitation for bleeding trauma patients [24–26], endothelin for chronic heart failure [27, 28] and more recently, a booster vaccine (MVA85A) intended to confer extra protection against tuberculosis [29, 30]. In all these cases, it would be reasonable to conclude that conducting prospective SRs of the animal studies might have prevented expensive and unnecessary or risky clinical trials from proceeding; indeed this is often called for following the failure of a clinical trial, or the halting of a clinical trial that turns out to be dangerous [31].

In all the above cases however, there was concordance between the SRs of the animal and human data in terms of evidence of efficacy. Unfortunately this is not always the case, and it is the possibility of discordance that raises doubts about the benefits of conducting SRs of animal studies prior to clinical trials. For example, a clinical trial of probiotic supplementation found that it increased mortality in humans [32], but a retrospective SR of the animal data found no indication of any risk of mortality; in fact the animal studies found evidence of benefit. The authors concluded that if the SR of the animal studies had been conducted *prior* to the clinical trial, it would not have predicted the harmful effects of probiotic supplementation on humans [33].

This issue can be further explored using Perel et al.’s data [10]. These authors identified six interventions for which there was unambiguous SR evidence of a treatment effect for humans and then conducted SRs of the corresponding animal studies for the same six interventions. The quality of the animal experiments was judged to be poor across all six interventions. Based on the SR evidence they found that there was concordance or partial concordance between the animal and human data for three of the interventions (antenatal corticosteroids, thrombolytics and bisphosphonates). However, for the other three interventions there was discordance between the animal and human data: antifibrinolytics reduce surgical bleeding and the need for transfusion in humans [34] but the animal studies of antifibrinolytics were inconclusive [10]; corticosteroids benefit animals with brain injury [10] but increase mortality in humans with brain injury [35]; tirilazad reduces infarct volume and improves neuro-behavioural scores in animals with experimental stroke [10] but increases the risk of death and dependency in humans with stroke [36]. What would have happened if the SRs of these animal studies had been conducted prior to clinical trials? Because the SR of animal studies of antifibrinolytics found the data to be inconclusive, the (ultimately beneficial) clinical trials of antifibrinolytics may not have gone ahead. In the case of corticosteroids and tirilazad, it is to be hoped that the SRs would have alerted clinical scientists to the poor quality of the primary animal studies, leading them to conclude that the animal data were not sufficiently robust to provide a basis for clinical trials; if not, the trials would have proceeded and patients would have died. The point is that a SR is only as good as the studies it includes. Since animal studies are unable to reliably predict safety and efficacy in humans [37–41] it follows that SRs of animal studies will not be able to reliably predict safety and efficacy in humans either.

### Will translational rates increase once the quality of animal studies improves?

The poor quality of preclinical animal studies is widely acknowledged [1, 10, 42–44]. MR-H’s team, for example, attempted to use a robot reviewer to evaluate the quality of animal studies for a risk of bias assessment, but was unable to train the robot reviewer as high calibre animal studies do not exist [45]. This lack of scientific rigour complicates matters, making it difficult to draw conclusions about animal to human translation. For example, if an animal study reports positive results, but the quality of that animal study is poor (as in the case of corticosteroids and tirilazad, in Perel et al.’s study above [10]), it is unclear whether those positive findings are ‘true’, or simply an artefact of poor study design. Many are of the view that improvements in scientific rigour and reporting will ultimately reveal greater concordance between animal and human data, but at present there is no evidence to support this view. In the field of stroke, for example, there have been concerted attempts to improve the quality of animal studies (e.g. [46, 47]), but these have not resulted in greater clinical translation [48–52]. This may be because the quality of the animal studies is still not sufficiently high, and/or it may be a result of the unpredictability that animal–human species differences introduce. As the quality of the animal studies in Perel et al.’s study [10] was judged to be poor for all 6 interventions, something other than study quality appears to have influenced the observed variation in concordance between animal and human data. A recent scoping review of 90 papers assessing the concordance of animal to human studies revealed that concordance ranges from 0 to 100% [53]. Although the number of studies increased with time, the concordance rates remained in the same broad range. This could indicate that despite efforts to improve the quality of animal studies, the risk of bias is still too high to draw reliable conclusions, or that animal to human translation will always be unpredictable due to animal–human species differences [54–59]. Animal–human species differences should not be underestimated, yet their contribution to the poor predictivity of animal models is vastly under-explored in comparison with other aspects that impact on translation, such as risk of bias and inappropriate animal models [60].

### Is it feasible to conduct prospective SRs of animal studies?

To return to the idea of conducting SRs of animal studies prior to clinical trials, there is an added difficulty in the context of disease research, because animal and human studies frequently run concurrently. Although it is a widely held view that studies are first conducted in animals and that the results from these inform clinical research, in fact the law only requires animal studies to be conducted prior to clinical studies for *safety* testing, i.e. there is no legal requirement for animals to be used in basic research into drugs and diseases. An examination of the publication dates of animal and human studies for the same interventions [16, 17, 21] indicates that animal and human studies often run alongside each other rather than consecutively and that in several cases the animal studies continue after human studies have stopped. Table 1, drawing on data from Perel et al.’s study [10], shows that there are no cases in which clinical studies commenced only after animal studies were completed.

**Table 1:** Dates of animal and human studies for the same intervention, from Perel et al. [10] and reanalysed by Pound et al. [17]

	Publication dates of animal studies	Publication dates of human studies
Antifibrinolytics	1967–1997	1987–1998
Bisphosphonates	1991–2005	1995–1999
Corticosteroids	1975–2005	1972–2005
Tirilazad	1990–2004	1994–1997
Antenatal corticosteroids	1971–2004	1972–2002
Thrombolysis	1987–2005	1981–2002

If animal and human studies run concurrently, it begs the question of how necessary animal studies are to clinical research and progress. Table 1 suggests that clinical researchers do not wait for animal study findings before proceeding to conduct human studies. Furthermore, clinicians tend to cite clinical (rather than animal) studies when drawing up clinical guidelines [61] and anecdotal evidence suggests that medical ethics committees rarely look at animal data once human data are available. All of this points to a rather confused attitude to animal studies: tradition demands that they precede human studies, but in practice clinical scientists may ignore their findings.

### How is clinical knowledge actually produced?

Translational research appears to be less linear that traditionally assumed, and the boundaries between the bench and the bedside more fluid [62]. In the field of regenerative medicine for example, it has been observed that innovation is frequently clinically driven, with basic laboratory science often validating existing clinical knowledge, rather than informing clinical research as is usually imagined [63]. This has been the case in the field of stroke too; thrombolytics went straight to clinical trials following success with heart attack, so while animal studies were conducted to investigate dosage, they were not necessary to establish that thrombolytics could be helpful for human stroke [64–67] (indeed microdosing in humans now offers a potential alternative approach for establishing dosage [68]). We need to better understand what is going on here, i.e. how much of the preclinical research effort is spent on animal studies designed to inform or predict the human situation, and how much is focused on other activities such as drug repurposing or reverse translation [69]. It is important to gain a more accurate picture of how new knowledge is produced, as well as the relative contributions of clinical and preclinical research activities in contributing to this new knowledge. Martin et al. [63] argue that clinical experimentation is a crucial driver in producing new knowledge and translating it into routine practice. For the sake of patients, we need to understand which are the most effective research practices for clinical knowledge production, so that more resources can flow into such practices.

## Conclusion

SRs of animal studies are clearly valuable for a number of reasons; they enable scrutiny of the validity of the preclinical evidence, they raise awareness of poor study design and ultimately encourage improvements in scientific rigour and reporting, they provide transparency, and help prevent the unnecessary duplication of animal studies. However, while SRs of animal studies conducted prior to clinical trials would provide valuable evidence about the validity of the animal data, they would not necessarily be able to reliably predict the safety or efficacy of interventions when trialled in humans, due to the poor predictivity of the primary studies. Therefore calls for SRs of animal studies to be conducted prior to clinical trials need to have a health warning; they are valuable for assessing the quality of the preclinical evidence, but do not necessarily have predictive validity, meaning that prospective SRs of animal studies will not reliably safeguard those taking part in clinical trials. We argue further, that it may well be impossible to conduct prospective SRs of animal studies, since this would require that animal studies are completed prior to clinical studies, which does not always appear to be the case. Finally, we suggest that it is time to assess the relative contributions of animal and human research in order to better understand how clinical knowledge is actually produced.

## Data Availability

Not applicable

## Abbreviations

SR: systematic review
M R-H: Merel Ritskes-Hoitinga
